# Supplementary material for: A relationship between Autism-Spectrum Quotient and face viewing behavior in 98 participants
Source: PLoS One. 2020 Apr 30;15(4):e0230866. doi: 10.1371/journal.pone.0230866 (PMC7192493; doi:10.1371/journal.pone.0230866)
Supplement: Attachment — Submitted filename: Response_to.docx [file pone.0230866.s001.docx]

General

1. *Please ensure that your manuscript meets PLOS ONE's style requirements, including those for file naming. The PLOS ONE style templates can be found at* [*http://www.journals.plos.org/plosone/s/file?id=wjVg/PLOSOne_formatting_sample_main_body.pdf*](http://www.journals.plos.org/plosone/s/file?id=wjVg/PLOSOne_formatting_sample_main_body.pdf) *and* [*http://www.journals.plos.org/plosone/s/file?id=ba62/PLOSOne_formatting_sample_title_authors_affiliations.pdf*](http://www.journals.plos.org/plosone/s/file?id=ba62/PLOSOne_formatting_sample_title_authors_affiliations.pdf)

**The formatting has been corrected.**

2. *We note that you have stated that you will provide repository information for your data at acceptance. Should your manuscript be accepted for publication, we will hold it until you provide the relevant accession numbers or DOIs necessary to access your data. If you wish to make changes to your Data Availability statement, please describe these changes in your cover letter and we will update your Data Availability statement to reflect the information you provide.*

**Data Dryad tell us**

Your dataset "A relationship between Autism-Spectrum Quotient and face viewing behavior in 98 participants" (doi:10.5061/dryad.zpc866t5c) has been successfully submitted. Your dataset will now remain private until your related manuscript has been accepted. At that point we will begin the curation process.

For private access during this review period, you may share your unpublished dataset using this temporary link: [https://datadryad.org/stash/share/lzLEL4oSmm6kuiI_6FphW1w7zAphQAo-0HCqHyXYV_0](https://urldefense.proofpoint.com/v2/url?u=https-3A__datadryad.org_stash_share_lzLEL4oSmm6kuiI-5F6FphW1w7zAphQAo-2D0HCqHyXYV-5F0&d=DwMFaQ&c=ZQs-KZ8oxEw0p81sqgiaRA&r=VbFWTztXfc-vRzzAGz_fmHhZV5hjoDUmhep-ica6IAU&m=NAK4PsV9iyuSPKSccbFSHdEz3E1FF8DtZr0BMNxJR30&s=4f2xO7TL-eBHPgzjeGtlIUD-rveXt35A9UAh3YTyH2Q&e=).

Your dataset has been assigned a unique identifier, called a DOI (doi:10.5061/dryad.zpc866t5c). This DOI may be provided to your journal, but it will not work until your submission has been approved by Dryad curators.

3. *We note that Figures 1,2,4 and 6includes an image of a [patient / participant / in the study].  As per the PLOS ONE policy (*[*http://journals.plos.org/plosone/s/submission-guidelines#loc-human-subjects-research*](https://urldefense.proofpoint.com/v2/url?u=http-3A__journals.plos.org_plosone_s_submission-2Dguidelines-23loc-2Dhuman-2Dsubjects-2Dresearch&d=DwMGaQ&c=ZQs-KZ8oxEw0p81sqgiaRA&r=VbFWTztXfc-vRzzAGz_fmHhZV5hjoDUmhep-ica6IAU&m=mdzJZYeGdFt1-H29eUJE9SGyu1pnLrz3QEVZxwUjZwE&s=3lZUUs7dUn2AEnnM1V7UJRnAH3OgE2YX3JxCgBBbKIg&e=)*) on papers that include identifying, or potentially identifying, information, the individual(s) or parent(s)/guardian(s) must be informed of the terms of the PLOS open-access (CC-BY) license and provide specific permission for publication of these details under the terms of this license. Please download the* [*Consent Form for Publication in a PLOS Journal*](https://urldefense.proofpoint.com/v2/url?u=http-3A__journals.plos.org_plosone_s_file-3Fid-3D8ce6_plos-2Dconsent-2Dform-2Denglish.pdf&d=DwMGaQ&c=ZQs-KZ8oxEw0p81sqgiaRA&r=VbFWTztXfc-vRzzAGz_fmHhZV5hjoDUmhep-ica6IAU&m=mdzJZYeGdFt1-H29eUJE9SGyu1pnLrz3QEVZxwUjZwE&s=ykpt9N6Idom-Z5Ch1VkoYOc3X_rrDvcAsGprmxp0r1Y&e=) *(*[*http://journals.plos.org/plosone/s/file?id=8ce6/plos-consent-form-english.pdf*](https://urldefense.proofpoint.com/v2/url?u=http-3A__journals.plos.org_plosone_s_file-3Fid-3D8ce6_plos-2Dconsent-2Dform-2Denglish.pdf&d=DwMGaQ&c=ZQs-KZ8oxEw0p81sqgiaRA&r=VbFWTztXfc-vRzzAGz_fmHhZV5hjoDUmhep-ica6IAU&m=mdzJZYeGdFt1-H29eUJE9SGyu1pnLrz3QEVZxwUjZwE&s=ykpt9N6Idom-Z5Ch1VkoYOc3X_rrDvcAsGprmxp0r1Y&e=)*). The signed consent form should not be submitted with the manuscript, but should be securely filed in the individual's case notes. Please amend the methods section and ethics statement of the manuscript to explicitly state that the patient/participant has provided consent for publication: “The individual in this manuscript has given written informed consent (as outlined in PLOS consent form) to publish these case details”.*

**These figures do not contain case details or images of patients/participants in the study, but rather stimulus images an actor recorded for the study. All actors shown have signed consent forms for publication of their likeness, and these have been uploaded to the website.**

4. *We note that Figure 4 in your submission contains copyrighted images. All PLOS content is published under the Creative Commons Attribution License (CC BY 4.0), which means that the manuscript, images, and Supporting Information files will be freely available online, and any third party is permitted to access, download, copy, distribute, and use these materials in any way, even commercially, with proper attribution. For more information, see our copyright guidelines:* [*http://journals.plos.org/plosone/s/licenses-and-copyright*](http://journals.plos.org/plosone/s/licenses-and-copyright)*. We require you to either (1) present written permission from the copyright holder to publish these figures specifically under the CC BY 4.0 license, or (2) remove the figures from your submission.  In the figure caption of the copyrighted figure, please include the following text: “Reprinted from [ref] under a CC BY license, with permission from [name of publisher], original copyright [original copyright year].” If you are unable to obtain permission from the original copyright holder to publish these figures under the CC BY 4.0 license or if the copyright holder’s requirements are incompatible with the CC BY 4.0 license, please either i) remove the figure or ii) supply a replacement figure that complies with the CC BY 4.0 license. Please check copyright information on all replacement figures and update the figure caption with source information. If applicable, please specify in the figure caption text when a figure is similar but not identical to the original image and is therefore for illustrative purposes only.*

**A consent form has been uploaded.**

**Reviewer 1:**

1. *~ASD is not defined. In the Introduction, please define this acronym and provide a brief description of this condition, for non-expert readers.*

**ASD is now defined in the first sentence of the introduction:
“A propensity for individuals with Autism Spectrum Disorder (ASD) to avoid eye contact during social interactions was noted as early as 1943 (1)”**

2. *~The introduction provides a succinct and direct rationale for examining the influence autistic traits have upon face viewing behaviour. However, I would suggest you acknowledge the prior research studies that have also examined the influence of autistic traits upon face viewing behaviour, for balance and completeness. One example of this work is Vabalas and Freeth (2015, Journal of Autism and Developmental Disorders).*

**We thank the reviewer for bringing this very relevant work to our attention. We now cite it in the Introduction as follows:**

**"A previous study with 36 participants interacting face-to-face with an experimenter found a relationship between autistic traits and visual exploration during social interactions (14)."**

*~The Introduction provides a rationale for the influence of autistic traits upon face viewing; however, no rationale or justification is presented for why or how this may differ across viewing conditions and why two experiments were conducted using different types of stimuli. This needs to be addressed, perhaps either in the Introduction, or in a Discussion section that separates the Methods/Results of the two Experiments.*

**We thank the reviewer for this helpful suggestion. The two experiments were included in order to examine the effect of availability of different amount of social content. We now write in the Methods:**

**“Task instructions can dramatically influence the pattern of eye movements made by participants (6). Therefore, we presented two very different kinds of stimulus and task configurations. In the syllable experiment, participants reported the syllable spoken by a laboratory-recorded talker during a short 2-second video. In the sentence experiment, participants watched sentences excerpted from videos of conversations without an explicit task. While the syllable videos are tightly controlled, they are also relatively unnatural, with no social or semantic content. The sentence videos are less tightly-controlled, but the presence of social and semantic content make them more similar to the natural conditions one might encounter in face-to-face interactions (14)."**

*~The replicability of the Method could be improved by including more detail e.g., accuracy of calibrations, detail on any counterbalancing and/or randomisation, software used for data presentation/extraction. Please also double check the trial numbers/eye tracking time reported – these don’t quite add up based on my own calculations – perhaps it is the explanation of this that needs clarifying? Making these alternations would not only mean the Method could be directly replicated, but also allow for the quality of data to be evaluated by readers.*

**The trial number and eye tracking times are correct. In the syllable experiment, each of 240 trials lasts 3 seconds (2 second video + 1 second response), for a total of 12 minutes (3 seconds/per trial * 240 trials = 720 seconds = 12 minutes). In the sentence experiment, trial lengths and blocks varied. On average, each clip lasted approximately 23 seconds (23 seconds * 10 videos * 4 blocks = 15.5 minutes). The exact length of each clip and blocked varied slightly.**

**The other requested information has been added to the Methods, as follows:
“Stimuli were presented through Matlab with Psychtoolbox.”
 “The stimuli were randomized by speaker and syllable, then presented in the same order to all participants.”
“Movies were balanced between blocks by length and speaker gender, then the stimuli were presented in the same order to all participants.”**

3. *~Related to the above point, the Method is missing a few of the standardised sections one would expect to be included (e.g., Design and Participants). This made finding this information somewhat lengthy and more challenging – I would strongly advise you consider including these sections and adding in additional detail where necessary.*

**The methods have been reformatted and now has a section for “Design and Participants” that provides an overview and participant information as well as the more detailed sections for each experiment.**

4. ~Page 8 line 157: For Experiment 1 it is stated that the proportion of fixations included upon the lower half of the face were weighted by trial duration – this seems to contradict earlier information that all trials were 2 seconds in duration. I would advise this be clarified.

**Participants watched the video for 2 seconds, then had 1 second to respond for a total of 3 seconds per trial (3 seconds/per trial * 240 trials = 720 seconds = 12 minutes). To clarify this, we now write: “Total task time was 12 minutes”**

**We now clarify that it is weighted by the duration of the fixation within each trial, not the trial duration itself (which was always 2 seconds).**

5. ~Page 9 line 180: I have noticed a typo - “noise”

**Fixed.**

6. *~The field is now adopting the use of linear mixed effect models, which avoid issues with averaging across trials, and provides a more sensitive analysis, accounting for random variance associated with both participants and stimuli. I would suggest the authors consider adopting this technique to analyse the current data. I understand this is not a trivial task, however, there are now an abundance of very helpful and user friendly guides and information on how to conduct and interpret mixed model analysis.*

**We thank the reviewer for this excellent suggestion. We have not contructed a linear mixed effect model in R for both experiments. This information is now included in the Results.**

**Expt 1**
PC_Low = % time spent fixating the lower face;
AQ = participant’s score (across all subscales);
Talker = which speaker filmed the video for that trial;
Syllable = what syllable was in the video for that trial;
Code = Participant;
Run = what block the trial is from)

**Expt 2:**
P_Lower = % time spent fixating the lower face;
AQ = participant’s score (across all subscales);
Movie = video in trial;
Sub = Participant;
Run = what block the trial is from)

7. *~The analysis of the data focused upon examining proportion of fixation time spend within ROI subsuming the lower half of the face. In places, the authors appear to assume this means the remaining fixations were directed towards the upper half of the face (e.g., eye region). I accept that this is very likely, however, there is evidence that attention is directed to non-social information (e.g., background), when viewing social scenes (e.g., images that contain a face or person), in samples of participants with a diagnosis of autism. Therefore, I would advise the authors report data on fixations upon the upper half of the face too, if they are to conclude autistic traits are associated with reduced gaze towards this region, in addition to increased fixations upon the lower face/mouth region. This is critical for the interpretation of this data.*

**For the ROIs defined here, any fixation not in the upper face is in the lower face and vice versa. In the syllable task, a line was drawn across the full stimulus to divide the upper and lower face. In the sentence task, the faces were identified and then fixations within the faces were marked as either upper or lower face.**

**Few trials were spent outside the defined face box (sentence task - movie_data_70 below) or off either the eyes or mouth (clear_data_98 below). For both tasks, the median percent of fixation time spent outside the ROIs was 0%. As shown in the histogram below, even in trials with some fixations outside the face (greater than 0%), it was rarely the entire trial.**

8. *~I was surprised to read the effect of gender on viewing behaviour was analysed. What was the rationale for conducting this analysis? If this is exploratory, I would suggest it is important for the authors to explicitly state this. In addition, given no influence of gender was detected in the regression analysis, it is unclear why post hoc analyses were conducted.*

**We have deleted gender as both reviewers criticized it and it is not relevant to the results of our study. It was initially included because many studies in the autism literature that we examined included an analysis by gender.**

9. ~Throughout the report there are some very strong claims about the lack of understanding of the mechanisms that underpin individual variability in face viewing behaviour (e.g., page 14 line 293 – “the determinants of individual variability are unknown”). I question whether this accurately reflects the literature… I would suggest the authors might want to consider lessening the strength of these claims to acknowledge that some understanding of individual variation in face viewing behaviour has been developed, for example, the impact of anxiety and cultural background.

**In the discussion, we now write:**

**“These differences have been linked to personality differences and differences in cognitive ability, however, the determinants of this individual variability are not yet fully understood.”**

**We would be happy to include something on cultural differences as well, but cannot find anything in the literature showing that. Only one study we found looked at cultural differences in face processing (Rayner, et al 2007) that failed to find differences in fixation location during face processing between Chinese and American participants.**

10. ~There are a couple of sentences that could be clearer, so the authors may want to revisit the manuscript to increase precision in places e.g., “Our results demonstrate that individual’s face viewing behaviour can be predicted without actually measuring it…” Given eye tracking is a direct measure of face viewing behaviour, I am not sure what the intended meaning of this sentence is.

**This sentence was confusing and we have removed it. We meant that a questionnaire (AQ) could predict face viewing behavior.**

11. ~Page 15 line 303. The authors provide a fair explanation for the possible mechanisms that underpin autistic traits and the differential face viewing behaviour. I would suggest that for a more complete and balanced view the authors should also acknowledge the range of alternative explanations too.

**We now discuss that there could be common sources, such as cultural background, that result in both high AQ scores and face looking behavior, even though there is no direct relationship between AQ scores and face-looking behavior. We now write:**

**“It is also possible another factor might result in both high AQ scores and increased fixation to the mouth. For example, face viewing behavior has been linked to speech perception (Rennig, et al 2019), cultural background (Rayner, et al 2007), and personality (Perlman, et al 2009), but each only explains a portion of the variance in face viewing behavior.”**

12. ~In the Discussion, the authors make a good and valid point about the statistical power of previous studies. However, in the report there is no report of an a priori power analysis conducted for the current experiments. To strengthen this point and the manuscript, if a priori power analysis this was completed and contributed to the design of the current experiments (which the manuscript implies), this should be reported in the Method.

**We did run an a priori power analysis, but did not have a clear prediction for effect size in the literature. In clinical studies, effect sizes are quite large (ex. Klin, et al 2002; r=0.729 and r=-0.627), while in previous studies correlating face viewing with other behavioral measures in a nonclinical population, they’re more moderate (ex. r=0.34 in Gurler, et al 2014). Depending where within this range the true effect size is, an appropriate sample size was between 14 and 102. We selected a moderate effect size (r=0.4) which gave a suggested sample size of 71.**

**Reviewer 2:**

1. P.6, 116 - The gender distribution is not balanced. What are the implications of this imbalance for the analysis of gender effects?

**We have deleted gender as both reviewers criticized it and it is not relevant to the results of our study. It was initially included because many studies in the autism literature that we examined included an analysis by gender.**

2. .P.6, 118 - It would be good to provide additional details on the experimental setup. How far on average from the fixation crosshair did the stimuli appear? I assume stimuli have been always presented in the middle of the screen?

**Stimuli did always presented in the middle of the screen. We now add:

“Eye tracking was performed with a sampling rate of 500 Hz. Before each run of each task, a 9-target array was presented for eye-tracker calibration and validation. Three times within each run, participants fixated a centrally presented crosshair.”**

3. P.8, 155-159 - Why was a more precise analysis of looking preferences not adopted? Could the authors please justify their choice of analysis? I would anticipate that the measure to correlate with AQ should be the looking preferences towards the eyes. This would establish consistency with the autism literature and would also allow aligning the assessment of looking preferences in the two types of stimulus (short vs longer videos).

**Feature specific ROIs are common but have a number of drawbacks: *(1)* the experimenter must make an arbitrary decision as to what features constitute an ROI (for example, the peri-mouth region contains visual information about mouth movements due to the structure of facial musculature and could arguably be included), *(2)* unequal sizes between mouth and eye regions, and *(3)* data near the border of the ROI is unnecessarily discarded. We would have liked to use a data-driven method such as the PCA method outlined in Wegner-Clemens, et al 2019 (Journal of Vision). However spatial PCA is highly sensitive to small changes in a feature’s location and there are more small movements of the eyes or mouth from frame to frame (even after the face detection algorithm).**

**Ideally, we would like to apply a data-driven approach such as a spatial PCA (Wegner-Clemens, et al 2019 Journal of Vision), however this analysis is highly sensitive to small differences in alignment between images. This extra noise means that the PCs obtained do not explain as much of the variance for the sentence task as the syllable task, so we opted to use an ROI based technique instead for consistency.**

4. I think that dichotomising the screen provides a rather coarse measure of looking strategies. What if people have looked at the corners of the screen or at the clothes? I also wonder if the use of this measure in this study underlies the difference between the two experiments shown in Figure 6. Have the authors considered this possibility?

**We have considered this possibility and did a coarse analysis of time spent off the face before selecting our final ROIs as well as visualizing heat maps for the entire stimuli.**

**Few trials were spent outside the defined face box (sentence task - movie_data_70	below) or off either the eyes or mouth (clear_data_98 below). For both tasks, the	median percent of fixation time spent outside the ROIs was 0%. As shown in the	histogram below, even in trials with some fixations outside the face (greater than 0%),	it was rarely the entire trial.**

5. P. 9, 166-168 - How often did this happen?

**The face detection tool failed infrequently.**

6. P. 9, 176-186 - The heatmap visualisation method is self-explanatory, however, it is only applied at an individual level. It is difficult to know how representative is the example heatmaps provided for some of the participants. Could the authors provide a super-subject heatmap visualisation or alternatively make all the individual heatmaps available? The main concern is again the dichotomous measure of looking preferences in the first experiment, which does not convince me that all participants looked at faces only.

**A global heat map is not particularly meaningful – since there is such substantial individual variation, a global heat map will show a fixation pattern that is not representative of any specific participant’s face viewing behavior (Mehoudar, et al 2014). However, it**

**As shown above, only a small proportion of fixation time was spent outside the face.**

7. P.11, 223-226 - So is the correlation with looking at the upper part of the screen -0.19?

**Yes, the reviewer is correct; we now write; "as the upper and lower face ROIs were	complementary (all fixations fell in one or the other), this meant that r==-0.19"**

8. P.11, 230, Not sure why the engagement of participants was concluded here.

**This statement was based on subject self-report but we have removed it as it was not	quantified.**

9. P.12, 249-251 - So is the correlation with looking at the eyes -0.31. If not, is this difference from Experiment 1 responsible for the difference discussed in Figure 6?

**Yes, the reviewer is correct; we now write:**

**"as the upper and lower face ROIs were complementary (all fixations fell in one or the	other), this meant that r==-0.19"**

10. P.14, 279-288 - see earlier comment given the gender imbalance in the sample.

**Gender is now removed.**

11. P.15, 303-305. Have the authors examined their data considering subscales of AQ? Should the correlation be higher for the social subscale under this account?

**We have created another linear effects model that includes the subscales of the AQ. No subscale was significant on its own.**

12. Discussion. Some very interesting points were raised, however, I would welcome discussing this study more broadly. Could other constructs relevant to autism-like sensory sensitivities or intolerance to uncertainty also account for some variance? What would be the next steps for this very interesting study?

**We now discuss that there could be common sources, such as cultural background, that result in both high AQ scores and face looking behavior, even though there is no direct relationship between AQ scores and face-looking behavior. We now write:**

**“It is also possible another factor might result in both high AQ scores and increased fixation to the mouth. For example, face viewing behavior has been linked to speech perception (Rennig, et al 2019), cultural background (Rayner, et al 2007), and personality (Perlman, et al 2009), but each only explains a portion of the variance in face viewing behavior.”**

13. I could not locate the data in the Dryad repository.	**Data Dryad writes:**

Your dataset "A relationship between Autism-Spectrum Quotient and face viewing behavior in 98 participants" (doi:10.5061/dryad.zpc866t5c) has been successfully submitted. Your dataset will now remain private until your related manuscript has been accepted. At that point we will begin the curation process.

For private access during this review period, you may share your unpublished dataset using this temporary link: [https://datadryad.org/stash/share/lzLEL4oSmm6kuiI_6FphW1w7zAphQAo-0HCqHyXYV_0](https://urldefense.proofpoint.com/v2/url?u=https-3A__datadryad.org_stash_share_lzLEL4oSmm6kuiI-5F6FphW1w7zAphQAo-2D0HCqHyXYV-5F0&d=DwMFaQ&c=ZQs-KZ8oxEw0p81sqgiaRA&r=VbFWTztXfc-vRzzAGz_fmHhZV5hjoDUmhep-ica6IAU&m=NAK4PsV9iyuSPKSccbFSHdEz3E1FF8DtZr0BMNxJR30&s=4f2xO7TL-eBHPgzjeGtlIUD-rveXt35A9UAh3YTyH2Q&e=).

Your dataset has been assigned a unique identifier, called a DOI (doi:10.5061/dryad.zpc866t5c). This DOI may be provided to your journal, but it will not work until your submission has been approved by Dryad curators.
