# Supplementary material for: A relationship between Autism-Spectrum Quotient and face viewing behavior in 98 participants
Source: PLoS One. 2020 Apr 30;15(4):e0230866. doi: 10.1371/journal.pone.0230866 (PMC7192493; doi:10.1371/journal.pone.0230866)
Supplement: Attachment — Submitted filename: Response_to_R2.docx [file pone.0230866.s002.docx]

**Editor's Comment:**

*Please respond to the clarification points made by Reviewer 1. These are minor tidying up issues that will help provide balance. I am happy for both analyses to remain in the manuscript if you prefer, however, I leave the final decision up to you.*

Thank you for the opportunity to respond to Reviewer 1's comments. In Reviewer 1's review of the initial submission, they suggested a linear mixed effects (LME) model analysis of our data. We agree with the reviewer that this is the more appropriate analysis and it now constitutes the bulk of our reporting. We have decided to leave the simple correlation analysis in our manuscript as it takes up only one line of text and provides an intuitive measure of % variance explained (r^2^) which will be of value to readers.

**Reviewer 1's comments:**

*Reviewer #1: I thank the authors for responded to each of the reviewer comments. I have made some minor suggestions below that I would suggest are necessary before publication (following the response to reviewer comments).*

1. *It is great to see you have made raw data available, however, this would benefit from the inclusion of a meta-data file to explain what file and column represents. If you have any analysis scripts these would be useful to make available too, so readers can reproduce your analysis.*

**We thank the reviewer for this excellent suggestion. We have now uploaded both a meta-data file, titled *DataSummaryFile.docx* and an analysis script, titled *AQ_lme_trials.R* to the DataDryad repository entry, linked to the raw data for the study.**

2. *I suggested the authors consider using mixed effects models to analyse their data – this has been done, however, has been included as additional analyses as opposed to replacing the simple regression. The authors should choose one or the other technique to report. If you choose to include the mixed effects models, these should be reported in full. One reason perhaps against reporting the linear mixed effects models, is that I assume you conducted the power analysis for a simple regression? I apologise for this contradicting my initial suggestion, but this has only become apparent since your response to the original reviews re the a priori power analysis you conducted.*

**We thank the reviewer for the suggestion to use LMEs. As suggested by the Reviewer, we have greatly expanded our presentation of the results of these models, including two new Tables that show the entirety of the model output. We have also left in the results of the simple correlation analysis, as it takes up only one line of text and provides an intuitive measure of % variance explained (r^2^) which will be of value to readers.**

3. *I accept the authors response re the decision to focus analysis on only the lower half of the face. However, an explanation within the paper for this decision and evidence to support this decision is needed in the paper, else readers will likely question this.*

**We agree with the reviewer and now write in the *Discussion:***

"Our study used a dichotomous scheme to classify fixations into either the upper half or the lower half of the face. This was effective because the stimuli consisted almost entirely of a face, with the result that essentially all fixations were to the face (as shown in the individual subject fixation heat maps). For more naturalistic displays containing many objects, it would be important to use a different method for classifying fixations. While feature-specific ROIs are common in the literature, they have a number of drawbacks. First, the experimenter must make an arbitrary decision as to what features constitute an ROI. For example, the peri-mouth region contains visual information about mouth movements due to the structure of facial musculature and could arguably be included in a "mouth ROI". Second, difference ROIs may have unequal sizes, leading to differences in fixation time between ROIs, even under the null hypothesis of random looking behavior. Third, data near the border of the ROI is unnecessarily discarded. For these reasons, unbiased methods that examine the entirety of the fixation data are an attractive possibility for future studies (27). "

4. *It was surprising that the authors included reference to cultural differences in eye movement patterns during face processing, given they state they could not find evidence for this. There is research demonstrating these differences exist – if the authors choose to include reference to cultural differences, it would be appropriate to also refer to this research for balance. (e.g., see Blais et al., 2008).*

**We are confused by this comment. Our manuscript did not examine cultural differences in face viewing behavior and so it does not contain any data relevant to this topic. The last sentence of the *Discussion* mentioned other sources of inter-individual differences, citing a paper on cultural differences:**

"Personality (8) and cultural background (11) also contribute to differences in face viewing behavior."

**We have updated this sentence to include the citation to Blais et al. suggested by the Reviewer (another paper showing cultural differences in face viewing):**

"Personality (8) and cultural background (8,35) may also contribute to differences in face viewing behavior."

5. Both reviewers suggested a more in-depth discussion of the range of theoretical reasons for autistic traits influencing face scanning be included in the Discussion (Reviewer 1 point 12, Reviewer 2 point 12) – this point does not appear to have been addressed – the authors might want to consider including some discussion on this topic.

**We have added a paragraph to the *Discussion* to address this important point:**

"An aversion to fixating the eyes of a viewed face could arise from the perception of direct gaze as socially threatening(16). In this account, from an early age, individuals with ASD avoid direct eye contact because it activates neural circuits involved in threat detection(4,17,18). Greater degrees of autistic traits in healthy individuals might result in direct gaze being perceived as more threatening, resulting in greater avoidance of eye fixations and concomitant negative emotions. A relationship between autistic traits and the degree to which direct gaze is perceived as threatening can account both for some of the group differences in gaze viewing between ASD and controls, and for some individual differences within samples of control participants. Differences in face-viewing behavior might be due to a general relationship that spans both control and individuals with ASD, rather than a specific difference between group of ASD and control individuals. A related concept is the idea of "intolerance of uncertainty" (19), which may mediate the relationship between ASD and anxiety disorders (20). Anxiety disorders, including social anxiety, are found in up to 50% of autistic people (21,22). To the extent that faces evoke anxiety, people with ASD could avoid fixating the eyes (the most relevant part of the face for social communication) or avoid fixating the face entirely. Atypical sensory processing could also play a role in differences in face-looking behavior in people with ASD (23). "

**Reviewer 2's comment:**

1. *The authors have addressed all my comments. A minor issue is that a sentence in the response to point 6 was incomplete. However, I am happy to take the authors point about Mehoudar, et al. (2014) on board.*

**We are grateful to the Reviewer for their careful examination of the manuscript.**
